# Comparative Effectiveness of Lumen-Apposing Metal Stents and Plastic Stents for the Treatment of Pancreatic Walled-Off Necrosis: A Meta-analysis

**DOI:** 10.1093/jcag/gwab024

**Published:** 2021-08-07

**Authors:** Natalia Causada Calo, Kirles Bishay, Mohammad Yaghoobi, Yuhong Yuan, Jeffrey Mosko, Gary May, Yen-I Chen, Christopher Teshima

**Affiliations:** Division of Gastroenterology, The Ottawa Hospital, University of Ottawa, Ottawa, Ontario, Canada; Division of Gastroenterology, St. Michael’s Hospital, University of Toronto, Toronto, Ontario, Canada; Division of Gastroenterology, McMaster University, Hamilton, Ontario, Canada; Division of Gastroenterology, McMaster University, Hamilton, Ontario, Canada; Division of Gastroenterology, St. Michael’s Hospital, University of Toronto, Toronto, Ontario, Canada; Division of Gastroenterology, St. Michael’s Hospital, University of Toronto, Toronto, Ontario, Canada; Division of Gastroenterology, McGill University, Montreal, Québec, Canada; Division of Gastroenterology, St. Michael’s Hospital, University of Toronto, Toronto, Ontario, Canada

**Keywords:** Endoscopic ultrasound, Lumen-apposing metal stent, Metal stent, Pancreatic walled off necrosis, Pancreatitis, Peripancreatic fluid collection, Plastic stent

## Abstract

**Background:**

Plastic stents (PS), lumen-apposing metal stents (LAMS) and biflanged metal stents (BFMS) are used for initial drainage of pancreatic walled-off necrosis (WON). There are no strong evidence to support the use of LAMS/BFMS over PS, and prior systematic reviews lack comparative analyses and also lack both trial data and observational studies for WON efficacy outcomes. The aim of this study is to compare the efficacy and adverse events (AEs) in LAMS/BFMS versus PS in patients with pancreatic WON.

**Methods:**

A comprehensive search up to December 1, 2020, was performed. The primary outcome was clinical improvement after drainage. Secondary outcomes included AEs and technical failure. Pooled odds ratios (OR) with 95% confidence intervals (CI) were reported using random effects models. Heterogeneity was evaluated with the Cochrane *I*^2^ statistic. Subgroup and sensitivity analyses were performed. The quality of the evidence was assessed using Grading of Recommendations, Assessment, Development and Evaluations (GRADE).

**Results:**

Nine studies (one randomized controlled trial and eight observational) were included for the primary outcome including 493 patients treated with LAMS/BFMS and 514 with PS. LAMS/BFMS were associated with higher odds of clinical improvement compared with PS (OR 2.58; 95% CI 1.81, 3.68; *I*^2^ = 1%). This association remained robust in sensitivity analyses. The use of LAMS/BFMS was not associated with higher AEs (OR 1.22; 0.61, 2.46; *I*^2^ = 71%). There was no difference in technical failure (OR 1.06; 0.19, 6.00; *I*^2^ = 12%).

**Conclusions:**

LAMS/BFMS seem to result in better clinical outcomes compared with PS in patients with pancreatic WON, with comparable AEs and technical failure. Larger randomized controlled trials for this comparison are warranted.

## INTRODUCTION

Acute pancreatitis (AP) is the third most common gastrointestinal disorder in North America and accounts for 279,145 hospitalizations annually in the United States (1). Walled-off necrosis (WON) occurs in close to 20% of severe pancreatitis and when infected is associated with a mortality rate that varies between 8% and 39% (2). WON is defined as a collection with a radiologically identifiable capsule that typically develops after 4 weeks following the onset of necrotizing pancreatitis (3). Drainage is indicated in cases of infection, biliary or gastric outlet obstruction and abdominal pain (3).

In recent years, endoscopic ultrasound (EUS)-guided drainage of WON has replaced surgical interventions, given that it has been associated with lower morbidity and shorter admission to hospital (4–6). Initially, EUS-guided drainage with or without necrosectomy was mainly performed using double pigtail plastic stents (PS); however, fully covered metal stents, biflanged metal stents (BFMS) and in particular lumen-apposing metal stents (LAMS) have been rapidly replacing PS. LAMS have the advantage of better lumen apposition and have a larger diameter, which may facilitate more robust drainage and direct endoscopic necrosectomy. The evidence supporting the use of LAMS/BFMS over PS are limited, with some studies showing the superiority of LAMS over PS (7), and a recent randomized controlled trial (RCT) reporting equivalent efficacy between the two interventions (8). Overall, studies suggest that LAMS are indeed safe and effective for draining WON, with a similar rate of adverse events (AEs) when they are removed within 3 to 4 weeks (8,9). On the other hand, LAMS are substantially more expensive (10), and there is a paucity of RCT data comparing LAMS and PS for WON drainage and correlation with clinical patient-oriented outcomes.

To date, data supporting a specific approach for EUS-guided drainage of WON are scarce. This meta-analysis aims to better characterize the current evidence regarding the efficacy of LAMS/BFMS compared with PS for the treatment of WON.

## METHODS

### Overview

This systematic review was conducted and reported according to Preferred Reporting Items for Systematic Reviews and Meta-Analyses (PRISMA) statement recommendations. A detailed PRISMA checklist is provided in Supplementary Table 1. The protocol for this review was registered in PROSPERO (CRD 42020164630). The primary objective was to compare clinical improvement, defined as per the primary studies, during a follow-up period up to 12 months, between LAMS/BFMS and PS for the treatment of pancreatic WON. Secondary objectives were to compare the rate of AEs (bleeding, leak, perforation, stent burying and stent occlusion) and technical failure between the two treatments.

### Search Strategy

The databases MEDLINE, EMBASE, the Cochrane Central Register of Controlled Trials (CENTRAL) and the Cochrane Database of Systematic Reviews (CDSR) were searched from inception to December 1, 2020. The search strategy for EMBASE is reported in Supplementary Table 2. No publication date and language limits were applied. Clinical trials databases were searched through the International Clinical Trials Registry Platform of the World Health Organization (WHO), and directly in the UK National Research Register (NRR) Archive, both up to December 1, 2020.

The search strategy was built in collaboration with a research information specialist (Y.Y.) and is meant to include a broad population, including patients who also underwent combined drainage with percutaneous drains in both treatment arms (LAMS/BFMS and PS) as it is common practice.

### Eligibility Criteria

A study was eligible for inclusion if it was a comparative cohort study, quasi-experimental or randomized clinical trial, it was published in any language, either in an abstract or manuscript format, it compared the clinical success, as defined by the primary study investigators, between adult patients with WON (defined by imaging as a well-circumscribed necrotic collection) that had an indication for drainage because of infection, biliary obstruction, abdominal pain or gastric outlet obstruction and were treated with either LAMS/BFMS or PS, including combination with percutaneous drainage.

Studies were excluded if they were single arm, if the indication for endoscopic drainage was not clearly specified, if the study population had undergone surgical drainage before endoscopic drainage, and if the length of follow-up was shorter than 3 weeks.

### Study Selection

Following the removal of duplicates, citations were imported into Rayyan (M Ouzzani, Qatar Computing Research Institute, HBKU, Doha, Qatar). All abstracts were screened independently by two reviewers (N.C.C. and K.B.). In the case of disagreements, a third author (C.T.) reviewed the study, and consensus was achieved. The full-length texts of selected abstracts were retrieved and reviewed.

### Data Extraction and Study Quality

A data abstraction form was designed a priori to collect data from each included study. Two reviewers (N.C.C. and K.B.) independently extracted pre-established data points, in addition to performing assessments of risk of bias and the overall study quality. The risk of bias of the included studies was assessed independently by the two authors (N.C.C. and K.B.), using the Cochrane Collaboration’s tool (ROB1.0) to assess the risk of bias in randomized trials, which covers the following bias domains: selection bias (random sequence generation and allocation concealment), performance bias (blinding of participants and personnel, and blinding of outcome assessors), attrition bias (incomplete outcome data) and selective reporting (11). We considered studies to be at low risk of reporting bias if both efficacy and safety outcomes were reported. This was based on the consideration that both clinical success and AEs are key outcomes. For the non-randomized studies, we used the Newcastle–Ottawa scale (12). A score of ≥7, 4 to 6 and ≤3 was considered of high‑quality, medium‑quality and low‑quality study, respectively.

Disagreements were solved via discussion. Study authors were contacted for additional information when needed. As suggested in the Cochrane Handbook for Systematic Reviews of Interventions (11), this information was asked in form of open-ended questions, to reduce the risk of overly positive answers. Inter-reviewer discrepancies in data abstraction were resolved by consensus after input of a third author (C.T.). We used the Grading of Recommendations, Assessment, Development and Evaluations (GRADE) system to assess the certainty of the evidence according to study design, consistency, directness, imprecision and reporting bias for the reported outcomes in this review.

### Outcomes

The efficacy outcome was clinical improvement as defined in the primary studies (Table 1). The following data related to the primary outcome were also collected when available: number of procedures needed for debridement, requirement of percutaneous drainage, requirement of surgical intervention and mortality.

**Table 1.** Definition of the primary outcome in the studies included in the meta-analysis

Author [Ref], year	Definition of clinical improvement/treatment success
Abu Dayyeh, 2018	Resolution of WON without concomitant percutaneous drainage
Bang, 2019	Resolution of WON on CT scan in association with clinical resolution of symptoms at 6-month follow-up.
Bapaye, 2017	Symptom resolution and complete WON resolution on imaging at the end of the treatment period
Chen, 2019	Decrease of WON > 3 cm in 6 months without need for percutaneous drainage or surgery
Faisal, 2018	Complete collection resolution
Ge, 2018	Successful resolution of the WON
Mukai, 2015	Disappearance of symptoms or inflammation regardless of the collection size
Sahar, 2017	Ability to remove the percutaneous drain once the necrotic cavity had resolved as confirmed by CT scan without recurrence of fluid collection over the ensuing 4 weeks.
Siddiqui, 2017	Complete resolution of the WON cavity and resolution of the patient’s symptoms without need for reintervention at 6 months after the initial treatment, as seen on ambulatory clinic follow-up and cross-sectional imaging

CT, computed tomography; WON, walled-off necrosis.

The safety outcome was the incidence of AEs, including clinically significant bleeding, perforation, stent burying, occlusion or migration. Technical failure was also a secondary outcome and was defined as reported in the primary studies.

### Statistical Analysis

Odds ratios (OR) with their respective 95% confidence intervals (CI) were pooled and presented in Forest plots to compare clinical improvement with LAMS/BFMS and PS. We used DerSimonian and Laird random effects models to account for expected heterogeneity across study designs. Chi-square tests and *I*^2^ statistics were calculated as a measure of inter-study heterogeneity. *I*^2^ values of 0% to 30% were regarded as possibly unimportant, 30% to 50% as moderate heterogeneity, 50% to 75% as substantial heterogeneity and values > 75% considerable heterogeneity. We planned to use funnel plots as well as Egger’s and Begg’s tests to assess reporting bias when number of studies >10; however, only 9 studies were included in this review, so test for funnel plot asymmetry was performed. Several a priori subgroup analyses were planned. First, it was hypothesized that the type of metal stent could potentially influence outcomes, and hence a subgroup analysis comparing studies that used LAMS with those that used BFMS was planned. Second, we also conceived that several variables could influence the main outcome of interest, and therefore we performed several subgroup analyses, including the use of LAMS and PS with and without concomitant percutaneous drainage, length of follow-up above and below 6 months, and use of co-interventions (nasocystic drainage and hydrogen peroxide). We also conducted sensitivity analyses whereby fixed effects models were used rather than random effects models, or relative risk instead of OR, type of study design (RCT versus observational studies) and publication type (conference abstract versus published manuscript). Statistical analyses were performed RevMan 5.3 (Cochrane Collaboration).

## RESULTS

### Study Selection

**Figure 1.**
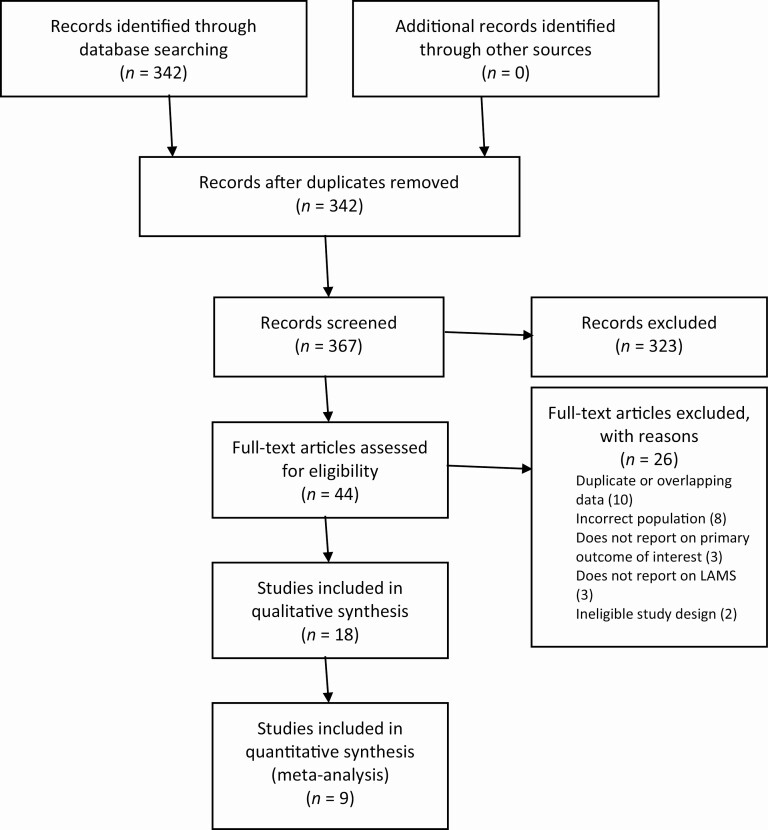
Preferred Reporting Items for Systematic Reviews and Meta-Analyses (PRISMA) flowchart.

A PRISMA flowchart of the search results and study selection process is presented in Figure 1. A total of 367 citations were identified by our search strategy. Of these, 44 full-text articles were reviewed. Nine studies were included in the meta-analysis of the primary outcome, with an additional nine studies included in the systematic review for primary and secondary outcomes.

### Study Characteristics and Quality

Baseline characteristics of the studies included in the meta-analysis are summarized in Table 2. All studies were published between 2015 and 2020, with most performed in North America. The vast majority were observational studies, and only one RCT was identified.

**Table 2.** Summary of studies included in the meta-analysis*

Author [Ref], year	Study design	Centres (*n*)	Plastic stent						LAMS/BFMS					
			Mean age (years)	Male (%)	Etiology: EtOH(%)/ stone(%)/ other(%)	Location of WON (%)	Stent type	Length of FU	Mean age (years)	Male (%)	Etiology: EtOH(%)/ stone(%)/ other(%)	Location of WON	Stent type	Length of FU
Abu Dayyeh, 2018	Obs	1	59.7	77.8	2.8/66.7/ 30.5	72.2 pancreatic/ peripancreatic only, 27.8 paracolic involvement	PS (7 and 10 Fr)	At least 12 weeks	52.7	77.6	25.9/39.7/ 34.5	84.5 pancreatic/ peripancreatic only, 15.5 paracolic involvement	LAMS (Axios 15 mm) (*n*. 45) 6 cm, 18 or 20 mm diameter fully covered metal stent, Niti-S; Taewoong Medical, Gyeonggi-do, South Korea) (*n*: 12)	At least 12 weeks
Bang, 2019	RCT	1	60.3	55.2	17.2/34.5/ 48.3	20.7 H, 79.3 B/T	PS (7 Fr)	24 weeks	55.8	64.5	29.0/19.4/ 51.6	29.0 H, 71.0 B/T	LAMS (cautery- enhanced Axios, 15 mm)	24 weeks
Bapaye, 2017	Obs	1	40.7	88.5	60.7/ 27.9/ 11.5	9.8 H, 86.9 B/T, 3.3 perihepatic	PS (7 Fr)	6–8 weeks	43.9	86.1	56.9/15.3/ 27.8	11.1 H/ 86.1 B/T, 2.8 perihepatic	BFMS (Nagi stent)	6–8 weeks
Chen, 2019	Obs	14	57.0	52.9	17.2/41.4/ 41.4	14.9 H, 66.7 B, 16.1 T	PS (size NA)	62.3 weeks	54.0	47.1	26.7/42.6/ 30.7	18.6 H, 66.7, 7.8 T	LAMS (Axios)	20.6 weeks
Faisal, 2018†	Obs	4	—	—	—	—	PS (size NA)	54 weeks	—	—	—	—	LAMS (type NA)	54 weeks
Ge, 2018†	Obs	1	—	—	—	—	PS (size NA)	At least 19.5 weeks	—	—	—	—	LAMS (type NA)	At least 11 weeks
Mukai, 2015	Obs	1	55.9	77.8	63.0/3.7/ 33.3	40.7 H, 33.3 B, 25.9 T	PS (7Fr)	NA	54.4	86.0	62.8/11.6/ 25.6	16.3 H, 46.5 B, 37.2 T	LAMS/BFMS (10- and 15- mm Axios; 16-mm Niti-S, and 12-mm Hanaro)	NA
Sahar, 2017	Obs	1	53.2	56.0	20.0/48.0/ 32.0	—	PS (7Fr, 3 cm)	~34 weeks	51.2	68.0	16.0/52.0/ 32.0	—	LAMS (Axios, 10 mm [56%] and 15 mm [44%])	~28 weeks
Siddiqui, 2017	Obs	2	56.3	64.2	34.0/34.0/ 32.0	17.9 H, 82.1 B/T	PS (10 Fr)	24 weeks	51.5	89.3	32.6/46.5/ 20.9	4.7 H, 95.3 B/T	LAMS (10 and 15 mm Axios)‡	24 weeks

B, body; BFMS, biflanged metal stents; EtOH, alcohol; Fr, French; FU, follow-up; H, head; LAMS, lumen-apposing metal stents; NA, not available; Obs, observational; PS, plastic stents; RCT, randomized controlled trial; T, tail.

^†^Baseline characteristics for plastic stents and LAMS/BFMS groups.

^‡^This study had a third group treated with self-expandable metal stents, but the data from this group were not extracted for this meta-analysis.

The summary of the quality of the studies is provided in Supplementary Table 3, and the summary of findings of the interventions and outcomes with the certainty of the evidence as per the GRADE approach is provided in Table 3.

**Table 3.** Summary of findings (GRADE, pending completion)

Outcomes	No. of participants (studies)	Certainty of the evidence (GRADE)	Relative effect (95% CI)	Anticipated absolute effects	
				Risk with plastic stents	Risk difference with LAMS/ BFMS*
Clinical improvement	1007 (8 observational studies, 1 RCT)	⨁⨁◯◯ LOW	**OR 2.58** (1.81, 3.68)	741 per 1000	**140 more per 1000** (97 more to 172 more)
Clinical improvement assessed with: CT scan and resolution of symptoms	60 (1 RCT)	⨁⨁◯◯ LOW	**OR 0.52** (0.04, 6.04)	966 per 1000	**30 fewer per 1000** (437 fewer to 29 more)
Number of endoscopic sessions	513 (5 observational studies)	⨁◯◯◯ VERY LOW	**MD −0.43** (**−**0.84 to **−**0.02)	—	—
Percutaneous drainage placement	443 (4 observational studies)	⨁◯◯◯ VERY LOW	**OR 0.68** (0.37, 1.24)	134 per 1000	**39 fewer per 1000** (80 fewer to 27 more)
Mortality	507 (7 observational studies)	⨁◯◯◯ VERY LOW	**OR 0.60** (0.22, 1.62)	39 per 1000	**15 fewer per 1000** (30 fewer to 23 more)
Any adverse event	891 (9 observational studies)	⨁◯◯◯ VERY LOW	**OR 1.22** (0.61, 2.46)	198 per 1000	**34 more per 1000** (67 fewer to 180 more)
Complication: bleeding	818 (8 observational studies)	⨁◯◯◯ VERY LOW	**OR 1.31** (0.46, 3.73)	44 per 1000	**13 more per 1000** (24 fewer to 103 more)
Stent obstruction	694 (5 observational studies)	⨁◯◯◯ VERY LOW	**OR 1.11** (0.26, 4.64)	102 per 1000	**10 more per 1000** (73 fewer to 243 more)
Stent migration	838 (7 observational studies)	⨁◯◯◯ VERY LOW	**OR 0.47** (0.22, 1.01)	52 per 1000	**27 fewer per 1000** (40 fewer to 0 fewer)
Perforation/peritonitis	506 (5 observational studies)	⨁◯◯◯ VERY LOW	**OR 2.12** (0.65, 6.90)	22 per 1000	**23 more per 1000** (8 fewer to 112 more)
Technical failure	888 (8 observational studies)	⨁◯◯◯ VERY LOW	**OR 1.06** (0.19, 6.00)	4 per 1000	**0 fewer per 1000** (4 fewer to 22 more)

GRADE Working Group grades of evidence—High certainty: We are very confident that the true effect lies close to that of the estimate of the effect; Moderate certainty: We are moderately confident in the effect estimate. The true effect is likely to be close to the estimate of the effect, but there is a possibility that it is substantially different; Low certainty: Our confidence in the effect estimate is limited. The true effect may be substantially different from the estimate of the effect; and Very low certainty: We have very little confidence in the effect estimate. The true effect is likely to be substantially different from the estimate of effect.

*The risk in the intervention group (and its 95% confidence interval) is based on the assumed risk in the comparison group and the *relative effect* of the intervention (and its 95% CI).

### Efficacy Outcomes

**Figure 2.**
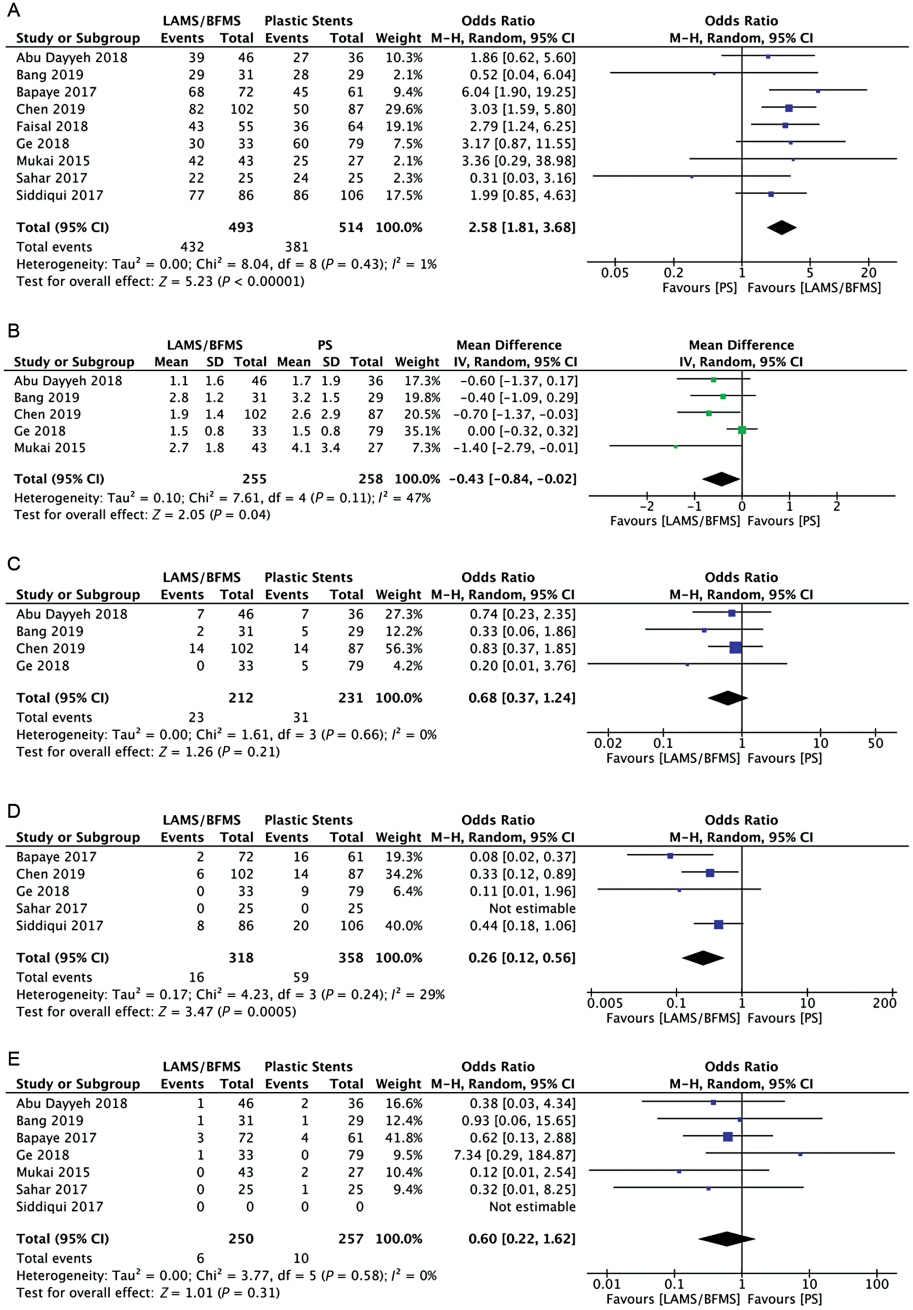
Efficacy outcomes. (a) Overall clinical improvement, (b) number of endoscopic sessions, (c) percutaneous drainage placement, (d) surgical intervention, and (e) mortality. BFMS, biflanged metal stents; CI, confidence interval; IV, inverse-variance; LAMS, lumen-apposing metal stents; M-H, Mantel-Haenszel; PS, plastic stents.

Nine studies were included in the meta-analysis for the outcome clinical improvement. The follow-up period was described in eight studies and ranged from 6 weeks (13) to 53 weeks (14). Most of the studies included in their clinical improvement or treatment success definition the resolution of the WON or a significant decrease in size, or either in the absence of concomitant interventions. The use of LAMS/BPMS was associated with clinical improvement (OR: 2.58; 95% CI 1.81, 3.68). The *I*^2^ was 1% demonstrating minimal heterogeneity in this analysis. (Figure 2a).

Five studies comprising 513 patients, 255 and 258 in the LAMS/BFMS and PS groups, respectively, reported the number of endoscopic sessions. The mean number of procedures was 2 for the LAMS/BFMS group and 2.6 for the PS group. Overall, the use of LAMS/BFMS was associated with a 57% pooled reduction in the number of endoscopic interventions (mean difference [MD] −0.43, 95% CI −0.84, −0.02). The *I*^2^ was 47% demonstrating moderate heterogeneity in this analysis (Figure 2b).

Four studies reported the need for percutaneous drainage, comprising 443 patients, 212 in the LAMS/BFMS group and 231 in the PS group. The need for percutaneous drainage was equivalent between the two interventions (OR 0.68; 95% CI 0.37,1.24). The *I*^2^ was 0% demonstrating no heterogeneity in this analysis. (Figure 2c).

Five studies reported the need for surgical interventions, comprising 676 patients, 318 corresponding to the LAMS/BFMS and 358 to the PS. The use of LAMS/BFMS was associated with a 74% reduction in the odds of requiring surgical interventions (95% CI 0.12, 0.56). The *I*^2^ was 29% demonstrating possibly unimportant heterogeneity in this analysis. (Figure 2d)

Seven studies reported mortality, comprising 507 patients, 250 and 257 in the LAMS/BFMS and PS groups, respectively. There was no difference in mortality between the groups (OR 0.60; 95% CI 0.22, 1.62). The *I*^2^ was 0% demonstrating no heterogeneity in this analysis (Figure 2e).

Nine studies met the inclusion criteria for the primary outcome of interest, but their data could not be included in the meta-analysis; they were included in the systematic review (Supplementary Table 4). Three studies reported greater clinical improvement with LAMS use (15–17), one showed a trend toward more clinical improvement with LAMS (18) and five did not observe any differences in clinical improvement (19–23).

### Adverse Events

Nine studies reported AEs, representing 891 patients, 427 in the LAMS/BPMS group and 464 in the PS group; there were no differences between the groups (OR 1.22; 95% CI 0.61,2.46). There was substantial heterogeneity in this analysis (*I*^*2*^ = 71%) (Supplementary Figure 1a). Clinically relevant bleeding was reported in eight studies, comprising 818 patients, 391 corresponding to the LAMS/BFMS group and 427 to the PS. There were no significant differences between the groups (OR 1.31: 95%CI 0.46,3.73). The heterogeneity between studies was substantial (*I*^*2*^ of 50%) (Supplementary Figure 1b).

Five studies reported data on stent obstruction, comprising 694 patients, 322 in the LAMS/BFMS group and 372 in the PS group. There was no difference in stent obstruction (OR 1.11: 95% CI 0.26, 4.64). There was considerable heterogeneity in this analysis (*I*^*2*^= 76%) (Supplementary Figure 1c).

Seven studies were included for the comparative analysis of stent migration, comprising 838 patients, 413 and 425 in the LAMS/BFMS and PS groups, respectively. There were no differences between the stents (OR 0.47: 95% CI 0.22,1.01). There was no heterogeneity in this analysis (*I*^*2*^ = 0%) (Supplementary Figure 1d).

Five studies reported data on the outcome perforation or peritonitis, comprising 506 patients, 233 and 273 in the LAMS/BFMS and PS groups, respectively. There were no differences between the groups (OR 2.12; 95% CI 0.65,6.90). There was possibly unimportant heterogeneity in this analysis (*I*^*2*^ = 16%) (Supplementary Figure 1e).

### Technical Failure

Eight studies were included in the meta-analysis of technical failure, representing 897 patients, 446 in the LAMS/BPMS group and 451 in the PS group. There were no differences in technical failure between the groups (OR 1.06; 95% CI 0.19, 6.00). There was possibly unimportant heterogeneity in this analysis (*I*^*2*^ = 12%) (Supplementary Figure 2).

### Subgroup Analyses

Several subgroup analyses were carried out for the primary outcome of clinical improvement. First, we compared studies that used LAMS to those that have used only BFMS or a combination of LAMS and BFMS. Only one study used BFMS alone (13), and another one a combination of both (24). In this subgroup analysis, the association between LAMS and clinical improvement persisted, and there were no significant differences between the subgroups (*P* = 0.14) (Supplementary Figure 3a).

Second, the length of follow-up was also considered as a potential influence on the primary outcome. Therefore, a subgroup analysis comparing studies with at least 6 months of follow-up to those with shorter length of follow-up was done. The association of LAMS/BFMS was also robust in this analysis, and there were no significant differences between groups (*P* = 0.37) (Supplementary Figure 3b).

Third, co-interventions such as the placement of nasocystic drainage and irrigation with hydrogen peroxide during necrosectomy could influence clinical efficacy. Therefore, subgroup analyses for these co-interventions were performed. Indeed, the association of LAMS/BFMS was also robust in these analyses for the use of nasocystic drainage (Supplementary Figure 3c) and hydrogen peroxide (Supplementary Figure 3d), and there were no significant differences between subgroups (*P* = 0.54 and *P* = 0.78, respectively).

### Sensitivity Analyses and Publication Bias

The association between LAMS/BFMS was also robust to sensitivity analyses including the comparison between type of publication (abstract format versus full-text publication) and study designs (RCT versus observational) (Supplementary Figure 4a and b). The findings for our primary outcome of clinical improvement were also robust to sensitivity analyses, as the OR did not change considerably with the exclusion of each study at a time, or with analysis using a fixed effects model (Supplementary Table 5). No evidence of publication bias for the primary outcome was observed by Egger’s or Begg’s tests or by visual inspection of the funnel plot (Supplementary Figure 5).

## DISCUSSION

In this first systematic review and meta-analysis comparing the efficacy of EUS-guided drainage with LAMS/BFMS versus PS for the treatment of symptomatic WON, we showed that the use of LAMS/BFMS, in comparison with PS, was associated with almost three times higher odds of clinical improvement. In addition, the type of stent was not associated with differences in AEs or technical failure. The quality of evidence as per GRADE was rated low mostly because most of the studies conducted thus far are observational.

Endoscopic transmural drainage has become the treatment of choice for the drainage of symptomatic or infected pancreatic WON and has replaced surgical interventions. An RCT has shown that open necrosectomy, in comparison to endoscopic drainage, is associated with higher morbidity (4). Furthermore, a recent randomized clinical trial also showed that endoscopic drainage, when compared with minimally invasive surgery, was associated with reduced major complications, lower costs, and better quality of life (6). For the endoscopic drainage of WON, the type of stent may influence the resolution of the collection and have subsequent clinical implications. The potential benefit of LAMS/BFMS over PS is due to their greater diameter that allows spontaneous passage of necrotic material into the gastric or duodenal lumen, potentially leading to faster clinical improvement and fewer necrosectomy sessions, all of which are relevant patient-oriented outcomes. Furthermore, cautery-enhanced LAMS are also generally preferred if available given their much simpler and faster deployment system. A recent systematic review and meta-analysis (25) pooled the rates of clinical success from studies that used LAMS and studies that used PS for the treatment of WON and found that the clinical success rate was 88.5% and 88.3% for studies that used LAMS and PS, respectively. The authors conclude that there were no differences between the two treatments in terms of clinical success and AEs. Our analysis, on the other hand, revealed that the use of LAMS/BFMS compared with PS was indeed associated with clinical improvement. Several reasons can explain this discrepancy: our inclusion criteria for both the study population and study design of the primary studies were different; we only included studies that compared outcomes in two or more groups of patients that had undergone treatment with different stents, rather than including single-arm studies. In addition, as opposed to the review by Babu et al. (25), we decided to include studies in which percutaneous drainage was used. The exclusion of these patients could potentially select cases with less severe or less complex necrotic collections, which are potentially easier to resolve with either stent and hence, is perhaps more difficult to see a difference in efficacy. Another systematic review and meta-analysis (9) compared the efficacy of LAMS versus PS for the drainage of pancreatic fluid collections and reported a greater clinical success with LAMS. However, this study did not separate the comparative efficacy analysis for the subgroup of patients with WON, which is the group considered to have the greatest benefit from LAMS to achieve source control in the case of infected necrosis. Unlike previous reviews on this topic, we included the only RCT (8) to date addressing this comparison. Interestingly, the RCT found no differences in clinical improvement between the two types of treatments, whereas the pooled estimate in the analysis of clinical benefit including only observational data suggests the contrary. Many underlying factors could potentially explain this difference. First, observational studies can be confounded by indication, which is an important limitation for an efficacy outcome. Also, most of the observational data were presented in an unadjusted fashion. Hence, the association between the use of LAMS/BFMS and clinical improvement is likely to be confounded and the association between LAMS/BFMS and clinical improvement could be overestimated. Second, it is possible that the RCT is underpowered to observe a true difference in clinical improvement between the groups given that their estimate was imprecise, and their primary outcome was a composite endpoint. Specifically, the RCT was powered to detect a difference of one procedure between LAMS and PS in achieving treatment success defined as radiological resolution and clinical resolution of symptoms at 6-month follow-up. Third, the protocol in the RCT differed from the practice in most of the observational studies for patients with collections over 120 mm as two drainage tracts were created with the same stent type. This may have contributed to the high rates of treatment success in both arms of the study, particularly when considering large and complex collections. It is also important to note that all AEs were observed in the LAMS arm three or more weeks following stent deployment, resulting in an amendment in the protocol that LAMS should be removed if there was radiological evidence of WON resolution at three weeks. Following this amendment, only two stent-related AEs were observed, suggesting that the safety profile of LAMS may be better when removed earlier on achieving clinical success. Furthermore, it is important to note that there was no standardization of length of time used for debridement across sessions, and this may have influenced the number of debridement sessions required to achieve clinical success. Finally, the definition of clinical improvement and length of follow-up varied across studies, which may have led to a different estimation of the outcome. This situation is a perfect scenario when performing a meta-analysis that can compensate for the lack of high-quality RCTs by including both observational studies and the only published RCT comparing the efficacy and safety of BFMS/LAMS versus PS for the treatment of patients specifically with WON, and by doing this, we were able to estimate associations for many secondary outcomes of interest. We also used GRADE to be able to provide a level for the quality of the evidence that our review presented.

The results of our meta-analysis should be interpreted with caution due to the nature of each systematic review which is dependent on the available data. For instance, most of the studies included in this review are observational, whereas this is an efficacy question better answered by randomized trials. However, our decision to include observational studies was necessary due to the paucity of trial data which is mainly because of the difficulty in performing such RCTs. Therefore, both observational studies and the only RCT to date were included and appropriate subgroup analyses were conducted to contrast the differences in the pooled estimates between the different study designs. On the other hand, the individual studies had different definitions for the primary outcome of clinical improvement or clinical success, which may have led to an erroneous representation of the true association between the treatment and the primary outcome. Furthermore, studies that used BFMS and LAMS were included, as opposed to including purely LAMS. This decision was based on the knowledge that the availability of LAMS and BFMS varies in different regions and having evidence on their efficacy could serve useful for decision-making. To mitigate this limitation, we performed a subgroup analysis comparing studies that used BFMS to those that only used LAMS and we did not find significant differences for the efficacy outcome. The follow-up period also varied across studies, which can also cause a difference in the estimation of the treatment efficacy. Hence, we performed a subgroup analysis comparing the outcome clinical improvement between studies with a follow-up period longer than 6 months to those with a follow-up shorter than 6 months and did not find significant differences between the groups. Lastly, other exposures such as co-interventions (i.e., irrigation with hydrogen peroxide in some cases and use of nasocystic drainage) could explain the residual heterogeneity of our main analyses, particularly in observational studies. Additional subgroup analyses for the placement of nasocystic drainages and use of hydrogen peroxide were performed (Supplementary Figures 3c and d). The heterogeneity was possibly unimportant, and the association of the LAMS/BFMS with clinical improvement was robust. Antibiotic use, on the other hand, was a co-intervention given in most of the studies to all patients irrespective of stent use.

## CONCLUSIONS

This systematic review and meta-analysis comparing the efficacy and safety of BFMS/LAMS versus PS for the drainage of WON suggest that BFMS/LAMS are associated with greater clinical improvement, while both stent groups seem to have equivalent safety. These findings must be interpreted with caution, and, after considering the level of evidence, it provides as depicted by the GRADE assessment. Nonetheless, the demonstration of greater clinical efficacy with BFMS/LAMS without compromising technical success or increasing AEs provides further evidence supporting the increasing clinical trend favouring the use of BFMS/LAMS, a trend that is further supported by the ease and efficiency of their placement. This may suggest that BFMS/LAMS should be considered, if available, as the first-line treatment for the initial EUS-guided drainage of WON.

## Supplementary Material

gwab024_suppl_Supplementary_Materials
