# Supplementary material for: Comparative Effectiveness of Lumen-Apposing Metal Stents and Plastic Stents for the Treatment of Pancreatic Walled-Off Necrosis: A Meta-analysis
Source: J Can Assoc Gastroenterol. 2021 Aug 7;5(2):68–78. doi: 10.1093/jcag/gwab024 (PMC8972216; doi:10.1093/jcag/gwab024)
Supplement: gwab024_suppl_Supplementary_Materials [file gwab024_suppl_supplementary_materials.docx]

**SUPPLEMENTARY MATERIAL**

**Supplementary Figure 1. Adverse events**

**Supplementary Figure 2. Technical failure**

**Supplementary Figure 3. Subgroup and sensitivity analyses**

**Supplementary Figure 4. Funnel plot**

**Supplementary Table 1. Prisma Checklist**

**Supplementary Table 2. Search strategy**

**Supplementary Table 3. Risk of bias assessment (detailed)**

**Supplementary Table 4. Summary of studies included in the systematic review for the primary outcome that were not included in the meta-analysis.**

**SUPPLEMENTARY FIGURES**

**Supplementary Figure 1. Adverse events**

**a. Any Adverse Event**

**
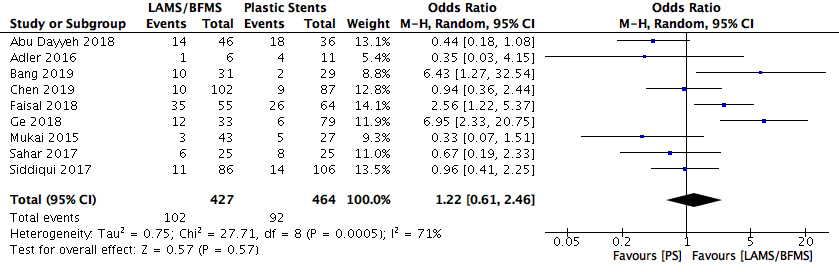
**

**b. Bleeding**

**
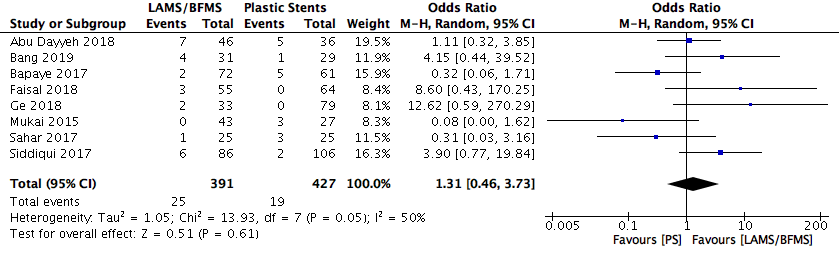
**

**c. Stent obstruction**

**
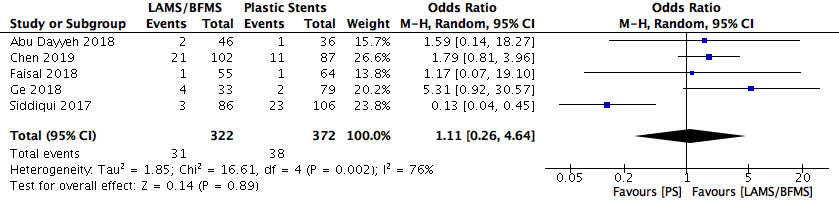
**

**d. Stent migration**

**
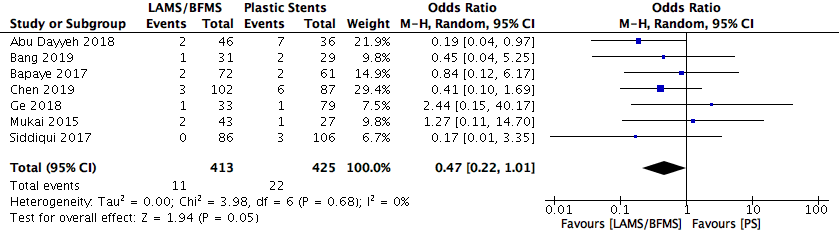
**

**e. Perforation/peritonitis**

**
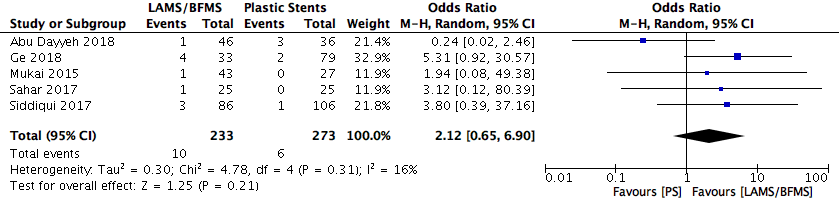
**

**Supplementary Figure 2. Technical failure**

**
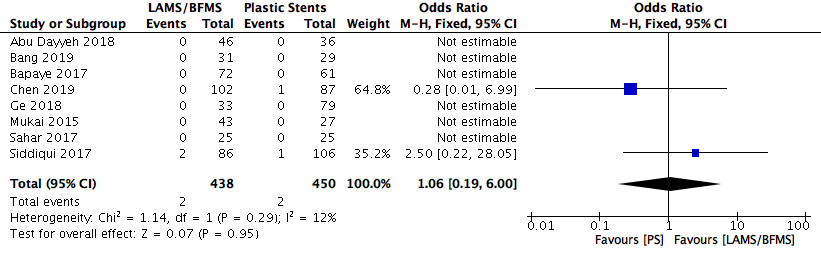
**

**Supplementary Figure 3**

1. **Subgroup analysis comparing the outcome clinical improvement between studies that used LAMS with those that used biflanged metal stents or a combination of BFMS and LAMS.**

**
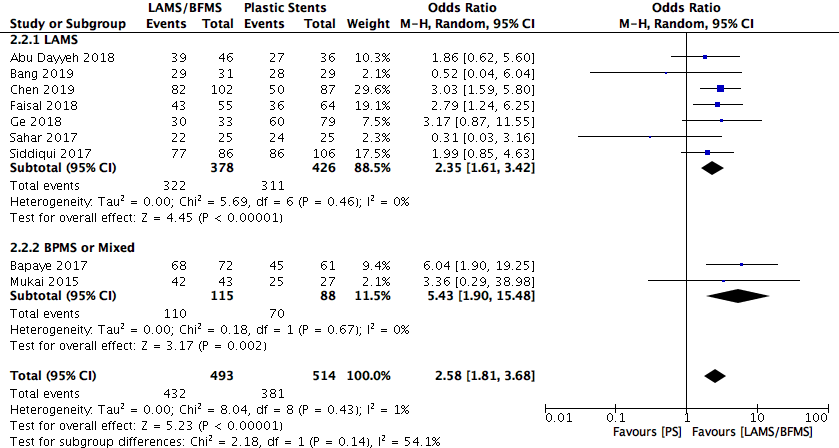
**

1. **Subgroup analysis comparing the outcome clinical improvement between studies with more than 6 months of follow up and those with less than 6 months of follow up**

**
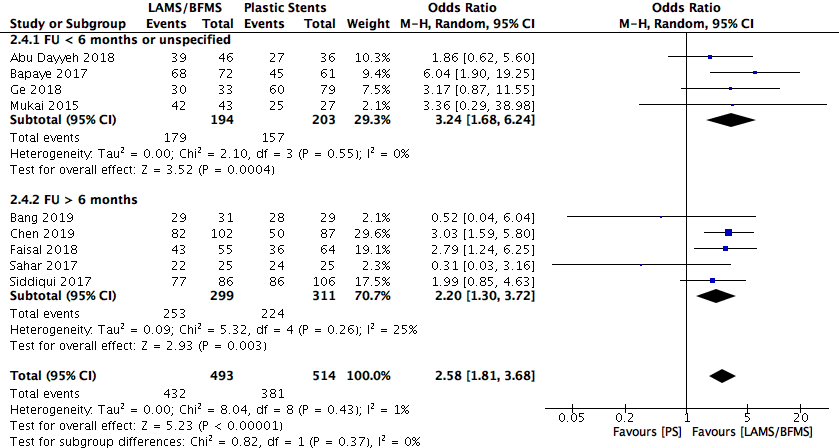
**

1. **Subgroup analysis comparing the outcome clinical improvement between studies where nasocystic drainage was placed and studies where it was not or it was not reported.**

**
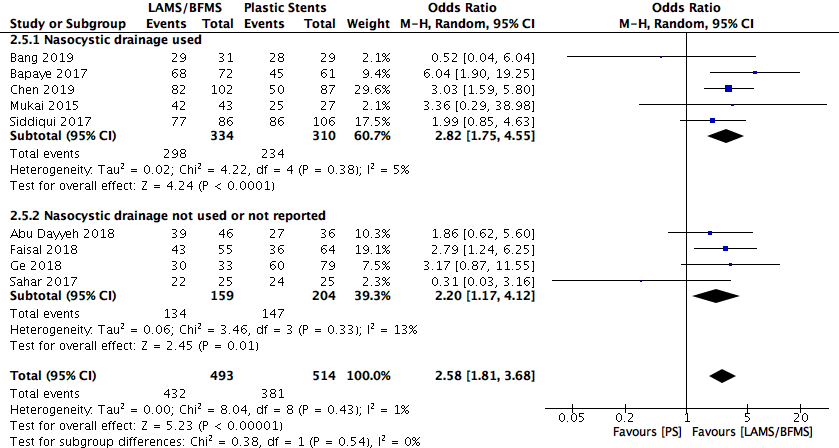
**

1. **Subgroup analysis comparing the outcome clinical improvement between studies that used hydrogen peroxide during necrosectomy and those that did not.**

**
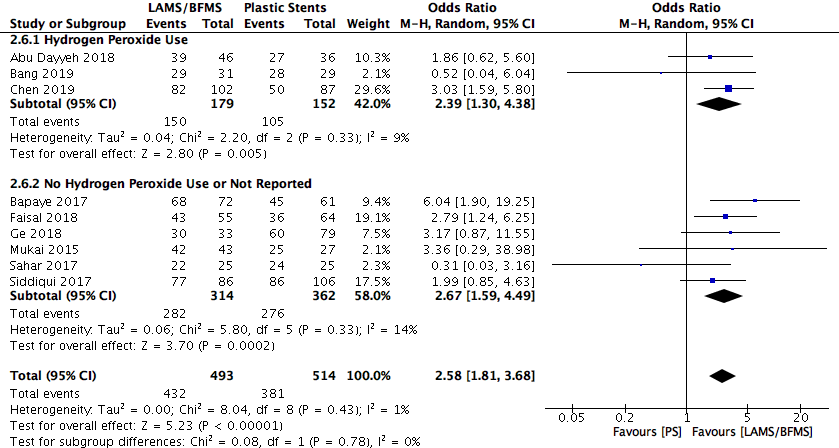
**

**Supplementary Figure 4:**

1. **Sensitivity analysis comparing the outcome clinical improvement between RCTs and observational studies**

**
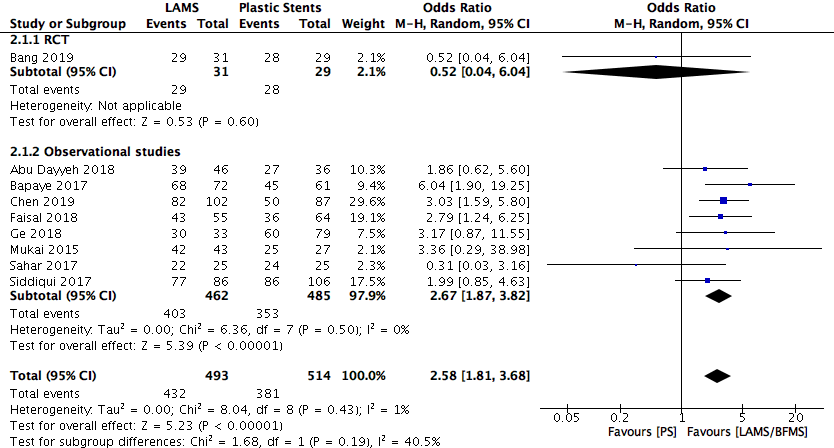
**

1. **Sensitivity analysis comparing the outcome clinical improvement between abstract format and full text publications**

**
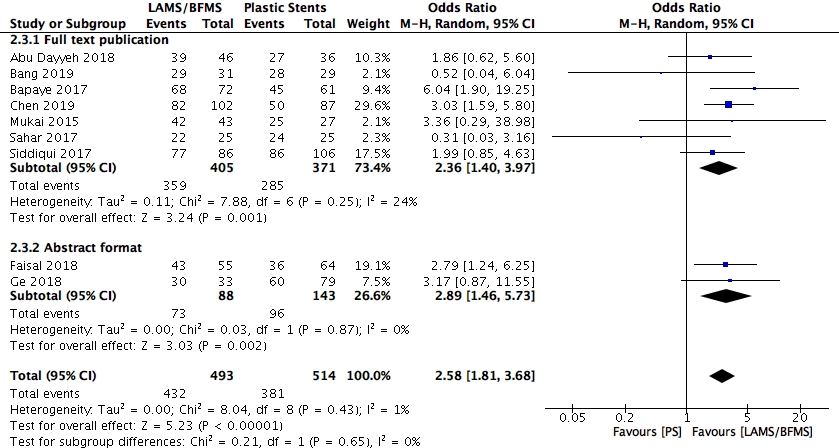
**

**Supplementary Figure 5. Publication bias (funnel plot)**

**
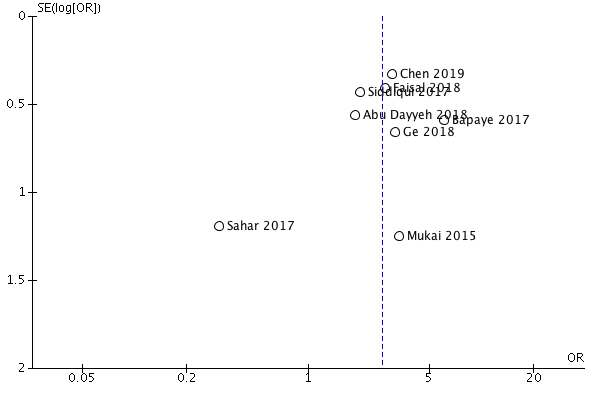
**

**SUPPLEMENTARY TABLES**

**Supplementary Table 1. Prisma Checklist**

| **Section/topic** | **#** | **Checklist item** | **Reported on page #** |
| --- | --- | --- | --- |
| **TITLE** | | |  |
| Title | 1 | Identify the report as a systematic review, meta-analysis, or both. | 1 |
| **ABSTRACT** | | |  |
| Structured summary | 2 | Provide a structured summary including, as applicable: background; objectives; data sources; study eligibility criteria, participants, and interventions; study appraisal and synthesis methods; results; limitations; conclusions and implications of key findings; systematic review registration number. | 2 |
| **INTRODUCTION** | | |  |
| Rationale | 3 | Describe the rationale for the review in the context of what is already known. | 4 |
| Objectives | 4 | Provide an explicit statement of questions being addressed with reference to participants, interventions, comparisons, outcomes, and study design (PICOS). | 5 |
| **METHODS** | | |  |
| Protocol and registration | 5 | Indicate if a review protocol exists, if and where it can be accessed (e.g., Web address), and, if available, provide registration information including registration number. | 5 |
| Eligibility criteria | 6 | Specify study characteristics (e.g., PICOS, length of follow-up) and report characteristics (e.g., years considered, language, publication status) used as criteria for eligibility, giving rationale. | 6 |
| Information sources | 7 | Describe all information sources (e.g., databases with dates of coverage, contact with study authors to identify additional studies) in the search and date last searched. | 5-6 |
| Search | 8 | Present full electronic search strategy for at least one database, including any limits used, such that it could be repeated. | Suppl Table 2 |
| Study selection | 9 | State the process for selecting studies (i.e., screening, eligibility, included in systematic review, and, if applicable, included in the meta-analysis). | 6-7, and Figure 1 |
| Data collection process | 10 | Describe method of data extraction from reports (e.g., piloted forms, independently, in duplicate) and any processes for obtaining and confirming data from investigators. | 6-7 |
| Data items | 11 | List and define all variables for which data were sought (e.g., PICOS, funding sources) and any assumptions and simplifications made. | 6-8 |
| Risk of bias in individual studies | 12 | Describe methods used for assessing risk of bias of individual studies (including specification of whether this was done at the study or outcome level), and how this information is to be used in any data synthesis. | 7-8 |
| Summary measures | 13 | State the principal summary measures (e.g., risk ratio, difference in means). | 8-9 |
| Synthesis of results | 14 | Describe the methods of handling data and combining results of studies, if done, including measures of consistency (e.g., I^2^) for each meta-analysis. | 8-9 |
| **Section/topic** | **#** | **Checklist item** | **Reported on page #** |
| Risk of bias across studies | 15 | Specify any assessment of risk of bias that may affect the cumulative evidence (e.g., publication bias, selective reporting within studies). | 9 |
| Additional analyses | 16 | Describe methods of additional analyses (e.g., sensitivity or subgroup analyses, meta-regression), if done, indicating which were pre-specified. | 9 |
| **RESULTS** | | |  |
| Study selection | 17 | Give numbers of studies screened, assessed for eligibility, and included in the review, with reasons for exclusions at each stage, ideally with a flow diagram. | Fig 1, 10-11 |
| Study characteristics | 18 | For each study, present characteristics for which data were extracted (e.g., study size, PICOS, follow-up period) and provide the citations. | Table 2 |
| Risk of bias within studies | 19 | Present data on risk of bias of each study and, if available, any outcome level assessment (see item 12). | Table 3 and Suppl Table 3 |
| Results of individual studies | 20 | For all outcomes considered (benefits or harms), present, for each study: (a) simple summary data for each intervention group (b) effect estimates and confidence intervals, ideally with a forest plot. | Fig 1 and 2, p. 10-14 |
| Synthesis of results | 21 | Present results of each meta-analysis done, including confidence intervals and measures of consistency. | 10-14 |
| Risk of bias across studies | 22 | Present results of any assessment of risk of bias across studies (see Item 15). | Suppl Table 3 |
| Additional analysis | 23 | Give results of additional analyses, if done (e.g., sensitivity or subgroup analyses, meta-regression [see Item 16]). | Suppl Table 5, S fig 2 |
| **DISCUSSION** | | |  |
| Summary of evidence | 24 | Summarize the main findings including the strength of evidence for each main outcome; consider their relevance to key groups (e.g., healthcare providers, users, and policy makers). | 14-15 |
| Limitations | 25 | Discuss limitations at study and outcome level (e.g., risk of bias), and at review-level (e.g., incomplete retrieval of identified research, reporting bias). | 17 |
| Conclusions | 26 | Provide a general interpretation of the results in the context of other evidence, and implications for future research. | 18 |
| **FUNDING** | | |  |
| Funding | 27 | Describe sources of funding for the systematic review and other support (e.g., supply of data); role of funders for the systematic review. | NA (no funding) |

*From:*  Moher D, Liberati A, Tetzlaff J, Altman DG, The PRISMA Group (2009). Preferred Reporting Items for Systematic Reviews and Meta-Analyses: The PRISMA Statement. PLoS Med 6(7): e1000097. doi:10.1371/journal.pmed1000097

For more information, visit: **www.prisma-statement.org**.

**Supplementary Table 2. Search strategy**

| Database: OVID Medline Epub Ahead of Print, In-Process & Other Non-Indexed Citations, Ovid MEDLINE(R) Daily and Ovid MEDLINE(R) 1946 to Present, Embase <1974 to 2020 December 1>, EBM Reviews - Cochrane Central Register of Controlled Trials <October 2020>, EBM Reviews - Cochrane Database of Systematic Reviews <2005 to December 1, 2020> |
| --- |
| Search Strategy: |
| -------------------------------------------------------------------------------- |
| 1 exp Pancreatitis, Acute Necrotizing/ (7837) |
| 2 exp acute hemorrhagic pancreatitis/ or exp hemorrhagic pancreatitis/ (4898) |
| 3 ((pancrea* or peripancrea*) adj10 (necros* or necroz* or walled off or WON)).tw,kw. (13906) |
| 4 ((pancrea* or peripancrea*) adj10 collect*).tw,kw. (10895) |
| 5 (pancreatitis adj5 (hemorrhag* or haemorrhag*)).tw,kw. (3609) |
| 6 or/1-5 (31294) |
| 7 exp stent/ or exp Stents/ (247285) |
| 8 stent*.af. (333477) |
| 9 or/7-8 (335508) |
| 10 6 and 9 (2386) |
| 11 (luminal* or lumen* or AXIOS or SPAXUS or LAMS or metal).tw,kw. (827565) |
| 12 (plastic or pigtail or DPS).tw,kw. (201220) |
| 13 10 and 11 and 12 (442) |
| 14 (exp animals/ or exp animal/ or exp nonhuman/ or exp animal experiment/ or animal model/ or animal tissue/ or non human/ or (rat or rats or mice or mouse or swine or porcine or murine or sheep or lambs or rabbit or rabbits or cat or cats or dog or dogs or cattle or bovine or monkey or monkeys or trout or marmoset$1 or basic research or cell lines or in vitro or animal model or canine).tw.) not (humans/ or human/ or human experiment/ or (human* or men or women or patients or subjects).tw.) (10627180) |
| 15 13 not 14 (440) |
| 16 (child/ or Pediatrics/ or Adolescent/ or Infant/ or adolescence/ or newborn/ or (baby or babies or child or children or pediatric* or paediatric* or peadiatric* or infant* or neonat* or newborn* or new born* or adolescen* or toddler*).tw.) not (adult/ or aged/ or (aged or adult* or elder* or senior* or men or women).tw.) (4210730) |
| 17 15 not 16 (433) |
| 18 remove duplicates from 17 (367) |

**Supplementary Table 3. Quality of the included studies**

**3a. Risk of bias assessment for observational studies**

| **Ref.** | **Cohort selection**  **(max 4)** | **Quotes**  **for cohort selection** | **Cohort comparability (Max 2)** | **Quotes**  **for**  **cohort comparability** | **Cohort assessment of outcome (max 3)** | **Quote for assessment of outcome** | **Comments** | **Total** |
| --- | --- | --- | --- | --- | --- | --- | --- | --- |
| Abu-Dayyeh 2018 | 4 | Retrospective cohort, assembled with records of consecutive adult patients (>18 years) at Mayo Clinic, Rochester, Minnesota, between January 1, 2010 and March 31, 2016. | 1 | Most baseline patient and WON characteristics, including size, location, and timing of intervention after onset of acute pancreatitis, were equivalent between groups (Table 1). Only the etiology of pancreatitis and use of EUS guidance differed significantly between the groups (both P < .01) | 2 | The 2 primary study outcomes were rates of WON resolution in the DPPS and LC-SEMS groups and percentage of patients who did not undergo subsequent endoscopic transmural necrosectomy sessions after endoscopic trans- mural drainage in each group. WON resolution was defined as complete clinical amelioration of index acute symptoms that triggered the intervention (resolution of the infection for infected WON or resolution of symptoms for symptomatic sterile WON) and radiographic resolution of the collection on follow-up cross-sectional imaging, without surgery. |  | 7 |
| Bapaye 2017 | 3 | BFMS be- came available for clinical use in 2012. Prior to 2012, MPS were the standard in our unit for all PFC drainage. Our data on MPS for WON in 61/66 patients over 8 years was presented at United European Gastroenterology (UEG) week 2012. Since 2012, 72 WON patients underwent EUTMD using BFMS over 36 months (2012–2014). These data were maintained in a prospective database | 1 | Both groups were comparable for age, cyst location, etiology of pancreatitis and indication for drainage. Mean WON size in the MPS group was larger than in the BFMS group (117.13 ± 30.08 vs 100.93 ± 32 mm), P < 0.05). | 1 | Unclear follow up time, minimal details for outcome assessment |  | 5 |
| Chen 2019 | 4 | This is an international, multicenter retrospective study involving 14 centers (12 in North America, 2 in Europe). Consecutive patients who underwent EUS-guided WON drainage with either LAMS or PS between January 2012 and August 2016 were included. Patients were identified using center-specific endoscopic or billing databases. Electronic chart reviews were performed to capture variables | 2 | Well matched Table | 1 | Patients with pancreatic pseudocysts, defined as an organized collection without solid components developing 4 weeks post-acute pancreatitis, and patients with less than 30 days follow-up were excluded. |  | 7 |
| Faisal 2018 | 2 | Retrospective chart review of patients undergoing endoscopic management of WOPN at four tertiary referral centers in the United States was performed. | 0 | No description beyond. "There was no statistically significant difference between groups in collection diameter or extent" | 0 | No description of Assessment of outcomes |  | 2 |
| Ge 2018 | 3 | Subjects included patients who underwent DEN for the management of symptomatic WON from 2003-2017. | 0 | No description, Table 1 is descriptive of combined data | 0 | No description of Assessment of outcomes |  | 3 |
| Mukai 2015 | 4 | A total of 70 patients with WON, 15 of whom were included in a previously published study [15], were treated by EUS-guided drainage and an additional endoscopic procedure (e. g. DEN), be- tween October 2006 and September 2013at the Tokyo Medical University Hospital. | 1 | All patient characteristics were similar be- tween the two groups, with the exception of the size of WON, which was greater in the BFMS group (105.6 ± 40.0 vs. 77.1 ± 33.4 mm; P = 0.003) | 1 |  |  | 6 |
| Sahar 2017 | 3 | Patients who had undergone DMD of WON between July 2011 and June 2016 were included in the study. | 2 | Baseline characteristics of patients were carefully matched for demographics, clinical, laboratory and imaging parameters between the LAMS and DPS groups. | 2 |  |  | 7 |
| Siddiqui 2017 | 4 | The endoscopy database at both Cornell and Thomas Jefferson University Hospital was queried for all patients who had undergone EUS-guided drainage/debridement of a pancreatic WON between November 2009 and May 2015. Only patients with follow-up of 6 months or more were included in the study. | 1 | significantly more males than females in LAMS group | 3 | The primary outcome of this study was to evaluate and compare the overall clinical success rate of the 3 different stents for the drainage/debridement of WONs, defined as complete resolution of the WON cavity and resolution of the patient’s symptoms without need for reintervention at 6 months after the initial treatment, as seen on ambulatory clinic follow-up and cross-sectional imaging. |  | 8 |

**Table 3b. Risk of bias assessment for the randomized trial**

| **Ref.** | **Risk assessment** | **Comment** |
| --- | --- | --- |
| ***Bang 2019*** |  |  |
| Random sequence generation (selection bias) | Low risk | Computer-generated. |
| Allocation concealment (selection bias) | High risk | Use of block randomization; opaque envelopes. |
| Blinding of participants and personnel (performance bias) | High risk | Intervention cannot be blinded. |
| Blinding of outcome assessment (detection bias) | Low risk | Outcome assessment was blinded. |
| Incomplete outcome data (attrition bias) | Low risk | Undetected. |
| Selective reporting (reporting bias) | Low risk | Undetected. |
| Other bias | Low risk | Undetected. |

**Supplementary Table 4. Summary of included studies in the systematic review**

| **Reference** | **Design** | **Characteristics of the study population** | **Intervention (n)** | **Comparison (n)** | **Outcomes assessed** | **Effect measure** |
| --- | --- | --- | --- | --- | --- | --- |
| Al-Azzawi 2017¶ | Cohort | Patients with peripancreatic fluid collections (pancreatic pseudocysts and necrosis combined)  Mean age: 52 y; 67% male  Main causes of pancreatitis: gallstone pancreatitis (40%), and  alcohol (28%)  WON represented 52% of the indications for the stent placement | Drainage with Axios stent (12) | Drainage with PS (42) | 1. Time to cyst resolution and stent removal. 2. Number of necrosectomies | 1. 81 days PS vs 48 days LAMS (P=0.01) 2. 26 patients in the pigtail group needed necrosectomy, and 5 patients in the Axios group |
| Ang 2016 | Cohort | Patients with peripancreatic fluid collections (pancreatic pseudocysts and necrosis combined)  Mean Age: 56y PS, 50y LAMS; 51% male  Main causes of pancreatitis: N/R  WON represented 37% of the indications for the stent placement | Drainage with Nagi stent (12) | Drainage with PS (37) | 1. technical and clinical success 2. need for repeat procedures 3. procedure related complications | 1. technical success 100% in both groups; clinical success 64.9% PS vs 91.7% LAMS (P=0.07) 2. 34.2% PS vs 6.3% LAMS (P=0.03) 3. 13.5% PS vs. 0% LAMS (P=0.18) |
| Brimhall 2018 | Cohort | Patients with peripancreatic fluid collections (pancreatic pseudocysts and necrosis combined)  Mean age: 48y PS, 47y LAMS; 65% male  Main causes of pancreatitis: gallstone pancreatitis (28%), and  alcohol (32%)  WON represented 74% of the indications for the stent placement | Drainage with Axios stent (97) | Drainage with PS (152) | 1. technical and clinical success 2. mean number of intervention/direct endoscopic necrosectomy 3. procedure related complications | 1. technical success 90.1% PS vs 92.8% LAMS (P=0.67); clinical success 90.1% PS vs 91.8% LAMS (P=0.54) 2. 1.9 PS vs 1.7 LAMS (P=0.93) 3. 17.8% PS vs. 24.7% LAMS (P=0.67) |
| Drepper 2019¶ | Cohort | Patients with peripancreatic fluid collections (pancreatic pseudocysts and necrosis combined)  Mean age: 48; 73% male  Main causes of pancreatitis: N/R  WON represented 25% of the indications for the stent placement | Drainage with LAMS (brand N/R) (23) | Drainage with PS (47) or SEMS (5) | 1. technical and clinical success 2. procedure related complications 3. re-intervention rate | 1. technical success 100% PS vs 100% SEMS vs 82.6% LAMS (P=0.008); clinical success 87.2% PS vs 82.6% SEMS vs 60.0% LAMS (P=0.281) 2. 2.1% PS vs. 40% SEMS vs. 17.4% LAMS (P=0.006) 3. 14.9% PS vs. 80% SEMS vs. 4.3% LAMS (P=0.028) |
| Fasullo 2018 | Cohort | Patients with peripancreatic fluid collections (pancreatic pseudocysts and necrosis combined)  Mean age: 51.2y PS, 50.4y LAMS; 59% male  Main causes of pancreatitis: gallstone pancreatitis (40%), alcohol (28%), triglyceride (4%)  % WON N/R | Drainage with Axios stent (12) | Drainage with PS (42) | 1. time to fluid collection resolution 2. requirement of subsequent necrosectomy | 1. 102 days PS vs 57 days LAMS (P=0.02) 2. 54% PS vs. 42% LAMS (P=0.24) |
| Kerdsirichairat 2017¶ | Cohort | Patients with WON  Mean age: N/R; Sex N/R  Main causes of pancreatitis: N/R | Drainage with LAMS (brand N/R) (53) | Drainage with PS (56) or SEMS (29) | 1. time to complete resolution 2. procedure related complications | 1. 47 days LAMS vs 86 days other (P=0.008) 2. 30.2% LAMS vs. 21.7% other (P=0.24) |
| Lang 2018 | Cohort | Patients with peripancreatic fluid collections (pancreatic pseudocysts and necrosis combined)  Mean age: 52.2y PS, 54.6y LAMS; 60% male  Main causes of pancreatitis: gallstone pancreatitis (32%), alcohol (5.8%), triglyceride (19%)  WON represented 22% of the indications for the stent placement | Drainage with Axios stent (19) | Drainage with PS (84) | 1. technical and clinical success 2. procedure related complications 3. unplanned repeat endoscopy | 1. overall technical success 99%; clinical success 96% PS vs. 94% LAMS 2. 12% PS vs 53% LAMS (P=0.0003) 3. 10% PS vs. 26% LAMS (P=0.07) |
| Sioulas 2019 | Cohort | Patients with peripancreatic fluid collections (pancreatic pseudocysts and necrosis combined)  Mean age: 59.1y; 73% male  Main causes of pancreatitis: N/R  WON represented 34% of the indications for the stent placement | Drainage with Axios stent (84) | Drainage with PS (13) | 1. technical and clinical success 2. procedure related complications | 1. technical success 100% PS vs. 96.4% LAMS (P=1.00); clinical success 92.3% PS vs. 95.1% LAMS (P=1.00) 2. 7.7% PS vs. 15.6% LAMS (P=1.00) |
| Wang 2019 | Cohort | Patients with peripancreatic fluid collections (pancreatic pseudocysts and necrosis combined)  Mean age: 46.6y; 65% male  Main causes of pancreatitis: gallstone pancreatitis (51%), alcohol (13%),  WON represented 19% of the indications for the stent placement | Drainage with Micro-Tech, Nanjing, China (70) | Drainage with PS (62) or SEMS (28) | 1. technical and treatment success 2. need for re-intervention | 1. technical success 93.5% PS vs 96.4% SEMS vs 94.3% LAMS (P = 1.00); treatment success 4.6% PS vs 85.2% SEMS vs 89.2% LAMS (P = 0.76) 2. 13.5% PS vs. 25.9% SEMS vs. 27.7 LAMS (P=0.161) |

¶ Denotes conference abstract

N/R=Not reported

LAMS=Lumen apposing metal stent

PS= Plastic stent

SEMS= Self expanding metal stent

**Supplementary Table 5.** Summary of subgroup and sensitivity analyses for the primary outcome

| **Subgroup** | **Studies** | **Participants** | **Statistical Method** | **Effect Estimate** |
| --- | --- | --- | --- | --- |
| **2.1** **Study design** | 9 | 1007 | Odds Ratio (M-H, Random, 95% CI) | 2.58 [1.81, 3.68] |
| 2.1.1 RCT | 1 | 60 | Odds Ratio (M-H, Random, 95% CI) | 0.52 [0.04, 6.04] |
| 2.1.2 Observational studies | 8 | 947 | Odds Ratio (M-H, Random, 95% CI) | 2.67 [1.87, 3.82] |
| **2.2** **Type of stent** | 9 | 1007 | Odds Ratio (M-H, Random, 95% CI) | 2.58 [1.81, 3.68] |
| 2.2.1 LAMS | 7 | 804 | Odds Ratio (M-H, Random, 95% CI) | 2.35 [1.61, 3.42] |
| 2.2.2 BPMS or Mixed | 2 | 203 | Odds Ratio (M-H, Random, 95% CI) | 5.43 [1.90, 15.48] |
| **2.3** **Type of publication** | 9 | 1007 | Odds Ratio (M-H, Random, 95% CI) | 2.58 [1.81, 3.68] |
| 2.3.1 Full text publication | 7 | 776 | Odds Ratio (M-H, Random, 95% CI) | 2.36 [1.40, 3.97] |
| 2.3.2 Abstract format | 2 | 231 | Odds Ratio (M-H, Random, 95% CI) | 2.89 [1.46, 5.73] |
| **2.4 Length of follow-up** | 9 | 1007 | Odds Ratio (M-H, Random, 95% CI) | 2.58 [1.81, 3.68] |
| 2.4.1 FU < 6 months or unspecified | 4 | 397 | Odds Ratio (M-H, Random, 95% CI) | 3.24 [1.68, 6.24] |
| 2.4.2 FU > 6 months | 5 | 610 | Odds Ratio (M-H, Random, 95% CI) | 2.20 [1.30, 3.72] |
